# Chimeric antigen receptor (CAR)-T-cell therapy in non-small-cell lung cancer (NSCLC): current status and future perspectives

**DOI:** 10.1007/s00262-020-02735-0

**Published:** 2020-10-06

**Authors:** Jingjing Qu, Quanhui Mei, Lijun Chen, Jianying Zhou

**Affiliations:** 1 Department of Respiratory Disease, Thoracic Disease Centre, College of Medicine, The First Affiliated Hospital, Zhejiang University, Hangzhou, Zhejiang 310003 People’s Republic of China; 2 State Key Laboratory for Diagnosis and Treatment of Infectious Diseases, National Clinical Research Center for Infectious Diseases, Collaborative Innovation Center for Diagnosis and Treatment of Infectious Diseases, College of Medicine, The First Affiliated Hospital, Zhejiang University, Hangzhou, Zhejiang 310003 People’s Republic of China; 3 Department of Intensive Care Unit, The First People’s Hospital of Changde City, Changde, Hunan 415003 People’s Republic of China; 4 Lung Cancer and Gastroenterology Department, Hunan Cancer Hospital, Affiliated Tumor Hospital of Xiangya Medical School of Central South University, Changsha, Hunan 410008 People’s Republic of China; 5 Stowers Institute for Medical Research, 1000 E 50th Street, Kansas City, MO 64110 USA

**Keywords:** CAR-T-cell therapy, Non-small-cell lung cancer, Immunotherapy, Future perspective, Tumor microenvironment

## Abstract

There has been a rapid progress in developing genetically engineered T cells in recent years both in basic and clinical cancer studies. Chimeric antigen receptor (CAR)-T cells exert an immune response against various cancers, including the non-small-cell lung cancer (NSCLC). As novel agents of immunotherapy, CAR-T cells show great promise for NSCLC. However, targeting specific antigens in NSCLC with engineered CAR-T cells is complicated because of a lack of tumor-specific antigens, the immunosuppressive tumor microenvironment, low levels of infiltration of CAR-T cells into tumor tissue, and tumor antigen escape. Meanwhile, the clinical application of CAR-T cells remains limited due to the cases of on-target/off-tumor and neurological toxicity, as well as cytokine release syndrome. Hence, optimal CAR-T-cell design against NSCLC is urgently needed. In this review, we describe the basic structure and generation of CAR-T cells and summarize the common tumor-associated antigens targeted in clinical trials on CAR-T-cell therapy for NSCLC, as well as point out current challenges and novel strategies. Although many obstacles remain, the new/next generation of CARs show much promise. Taken together, research on CAR-T cells for the treatment of NSCLC is underway and has yielded promising preliminary results both in basic and pre-clinical medicine. More pre-clinical experiments and clinical trials are, therefore, warranted.

**Electronic supplementary material:**

The online version of this article (10.1007/s00262-020-02735-0) contains supplementary material, which is available to authorized users.

## Introduction

Lung cancer is the most common malignancy worldwide, as well as the leading and second greatest cause of cancer-related deaths in men and women, respectively [1, 2]. Owing to the typically late appearance of symptoms, most patients present at the advanced stage, with 5-year survival rates of 10–20% [3]. There are two main types of this disease: small-cell and non-small-cell lung cancer (NSCLC). NSCLC accounts for approximately 85% of all diagnosed lung cancers, causing a large proportion of lung cancer-related deaths [4].

The most effective therapies for NSCLC are cytoreductive surgery, chemotherapy, molecular targeted therapy, and immunotherapy [5]. Recently, novel NSCLC treatments have been proposed, from indiscriminate, cytotoxic chemotherapy to more precise therapies [6]. Hellmann et al. [7] reported that the combination of nivolumab plus ipilimumab yielded more favorable results than nivolumab monotherapy in patients with advanced NSCLC expressing programmed death-ligand 1 (PD-L1). Additionally, tyrosine kinase inhibitors may serve as clinical therapy for metastatic NSCLC patients harboring epidermal growth factor receptor (EGFR)-enhancing mutations [8]. Despite advances in surgical therapy, chemotherapy, and radiotherapy during the past 20 years that resulted in the increased survival time in NSCLC patients, the prognosis of NSCLC has not substantially improved, owing to the tumor mutational burden and the heterogeneity of the disease [4, 9–11]. Thus, it is imperative to explore novel strategies to prolong patient survival time.

Recent progress in immunotherapy has been promising; however, to improve the clinical prognosis in patients, more immunotherapy-focused pre-clinical and clinical studies should be performed [12, 13]. In some cases, NSCLC is already being alleviated using immunotherapy. Modified-T-cell therapy, particularly that using chimeric antigen receptor (CAR)-T cells, has attracted growing interest in various solid tumors in recent years [14, 15]. Anti-CD19 CAR-T cells are approved by the US Food and Drug Administration for the treatment of hematological B-cell malignancies, and clinical trials have yielded encouraging results [16, 17]. Recent progress indicates that CAR-T-cell therapy is also a promising strategy for the treatment of NSCLC [18, 19].

In this review, we outline the advances in CAR-T-cell therapy for NSCLC, including the structure and generation of CAR-T cells, as well as the antigens targeted. Particularly, we concentrate on major challenges and future prospects of CAR-T-cell immunotherapy to combat NSCLC.

## The structure and generation of CAR-T cells

### The structure of CAR-T cells

**Fig. 1:**
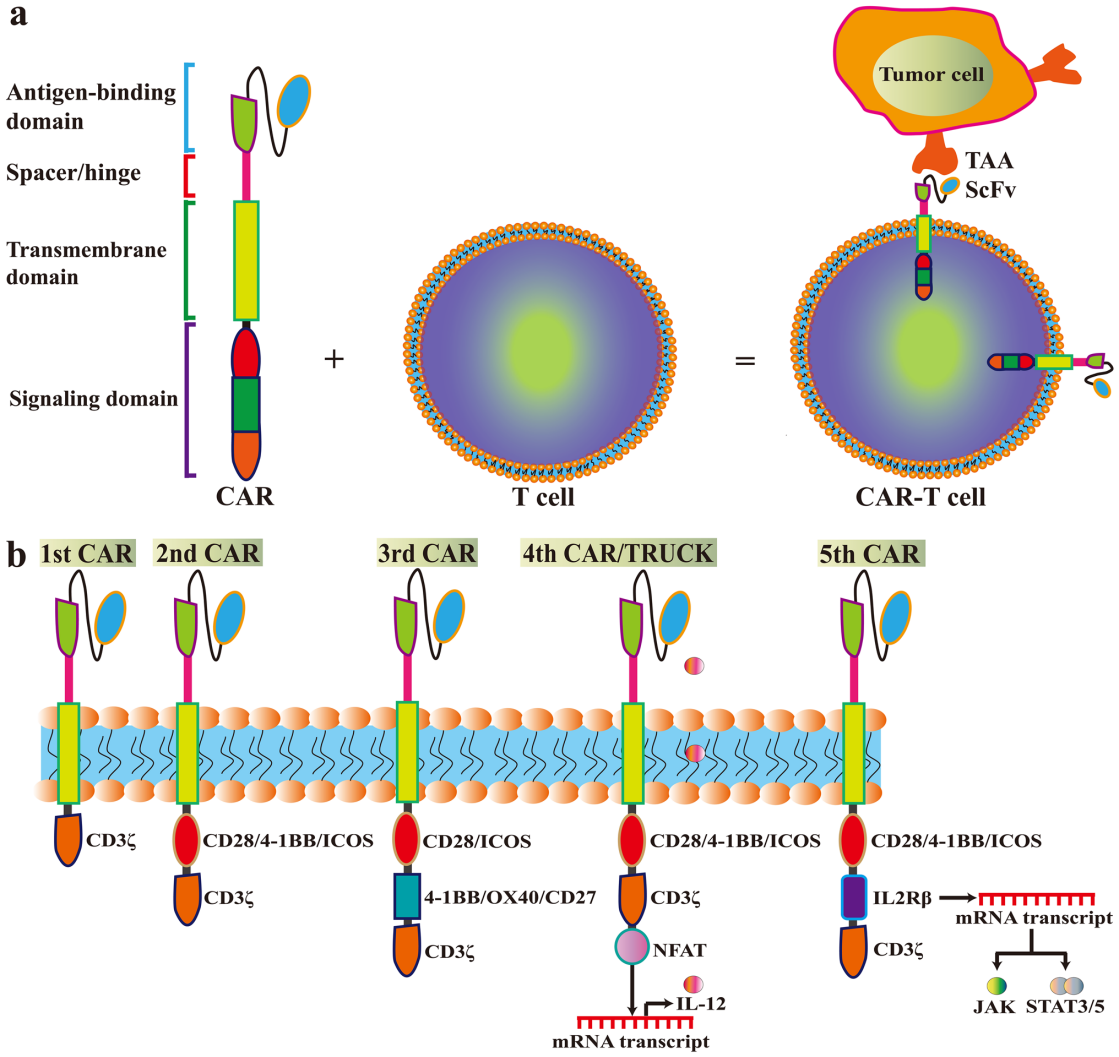
The structure and generation of chimeric antigen receptor (CAR)-T cells. **a** The CAR contains an extracellular antigen-binding domain, an extracellular spacer/hinge sequence motif, a transmembrane (TM) domain, and an intracellular signaling domain. The single-chain variable fragment (scFv) recognizes tumor-associated antigens (TAAs). The length of the spacer/hinge region can be adjusted to optimize the distance between CAR-T cells and targeted tumor cells for CAR signal transduction. The TM domain along with the spacer/hinge region, anchors the CAR to the cellular membrane. The signaling domain is an intracellular T-cell activation complex consisting of CD3ζ and many costimulatory molecules, which trigger antigen binding and modulate the downstream signaling cascade of T-cell activation. **b** In each new generation of CAR, signaling and applications are modified. The first generation of CARs included the scFv portion and the signaling endodomain of CD3ζ. The second generation of CARs added one costimulatory molecule (e.g., CD28, 4-1BB [CD137], and ICOS (inducible costimulator; CD278) to increase the persistence. The third generation of CARs contains CD3ζ and two costimulatory domains (e.g., CD28/ICOS and 4-1BB/OX-40/CD27). The fourth generation of CARs (also called TRUCKs) contains a nuclear factor of activated T cells (NFAT) domain, which induces a large amount of cytokines (e.g., IL-12). The fifth generation of CARs is based on the second generation, with the addition of a fragment of IL-2 receptor β (IL-2Rβ). The IL-2Rβ fragment can induce the production of Janus kinases (JAKs) and signal transducer and activator of transcription (STAT)-3/5 by mRNA transcription

CAR-T cells are genetically engineered T cells that express synthesized CAR vectors to specifically recognize and bind to antigens (such as CD19) on tumor cells [16, 20]. A CAR is an artificial fusion protein that contains an extracellular antigen-binding domain, an extracellular spacer/hinge sequence motif, a transmembrane (TM) domain, and an intracellular signaling domain (Fig. 1a). The single-chain variable fragment (scFv), which is the major element of the antigen-binding domain, can recognize tumor-associated antigens (TAAs) (Fig. 1a). Due to the presence of the scFv, CAR can specifically engage the target and trigger downstream signaling. The length of the spacer/hinge region can be adjusted to optimize the distance between CAR-T cells and targeted tumor cells for CAR signal transduction. The TM domain, along with the spacer/hinge region, anchors the CAR to the cellular membrane. The signaling domain is an intracellular T-cell activation complex consisting of a signal module CD3ζ (also called CD247) and many costimulatory molecules. These trigger antigen binding by modulating a downstream signaling cascade of T-cell activation (Fig. 1b). Costimulatory molecules include CD27, CD28, OX40 (CD134), 4-1BB (CD137), inducible costimulator (ICOS; CD278), and glucocorticoid-induced tumor necrosis factor (TNF) receptor-related protein (GITR) [14]. Among these, CD28, OX40, and 4-1BB are the most commonly used molecules.

### The generation of CAR-T cells

The safety and efficacy of CAR-T-cell therapy can be improved through optimizing CAR vectors [21]. The fifth generation of CARs is already being tested [15, 22]; the main differences between the generations of CARs are in the specific costimulatory molecules (Fig. 1b). The first generation of CARs includes only the CD3ζ signaling endodomain, fused to the extracellular scFv to confer modification and activation of T cells [23]. However, it had a short survival time and did not efficaciously triggered T-cell activation. To overcome this disadvantage, second-generation CARs commonly have an additional costimulatory molecule (e.g., CD28, 41BB, and ICOS) to increase the cells’ persistence [24]. Third-generation CARs contain CD3ζ and two costimulatory elements (e.g., CD27, CD28, 41BB, ICOS, and OX-40), which further increases and enhances their capacity to kill tumor cells [14, 25]. The fourth generation of CARs (also called T cells redirected for universal cytokine-mediated killing [TRUCKs]), instead of OX-40/CD27, contains a nuclear factor of activated T cells (NFAT) domain [26]. The NFAT domain induces a formation of a large amount of cytokines, especially interleukin (IL)-12, IL-15, and granulocyte–macrophage colony-stimulating factor, which modulates the anti-tumor microenvironment. The current, fifth generation of CARs, contains a fragment of IL-2 receptor β (IL-2Rβ) instead of OX-40/CD27. The IL-2Rβ fragment can induce the production of Janus kinases (JAKs) and signal transducer and activator of transcription (STAT)-3/5 [15, 27]. The fifth generation of CARs is under investigation for its safety and efficacy.

## NSCLC-associated antigens for CAR-T-cell therapy in clinical trials

T lymphocytes have been modified by synthetic CARs to recognize specific TAAs of numerous human malignant tumors, including those of solid tumors [28–30]. Among solid tumor types, a large proportion of CAR-T-cell research is focused on NSCLC [31]. The most commonly targeted antigens of NSCLC include EGFR, mesothelin (MSLN), mucin 1 (MUC1), prostate stem cell antigen (PSCA), carcinoembryonic antigen (CEA), PD-L1, CD80/CD86, inactive tyrosine-protein kinase transmembrane receptor (ROR1), and human epidermal growth factor receptor 2 (HER2). For a list of targeted antigens of NSCLC in clinical trials, see Table 1.

**Table 1:** Potential targeting antigens of NSCLC for CAR constructs in clinical trials

Targeting antigen	Intracellular costimulatory domain	Type of CAR	Estimated Enrollment	Phase	Sponsor	Indication	ClinicalTrials ID	Status	Posted date (M-D-Y)
EGFR	N/A	CXCR5 modified EGFR	11	I	Sun Yat-sen University, China	Advanced NSCLC	NCT04153799	Recruiting	11-6-2019
EGFR	CD28/4-1BB/CD3ζ	Anti-EGFR	60	I/II	Chinese PLA General Hospital, China	Chemotherapy refractory advanced solid tumors (include EGFR-positive NSCLC)	NCT01869166	Unknown	6-5-2013
MSLN	N/A	iCasp9M28z	179	I/II	Memorial Sloan Kettering Cancer Center, USA	Malignant pleural diseases (include NSCLC metastatic to the pleura)	NCT02414269	Recruiting	4-10-2015
MSLN	N/A	Anti-MSLN	15	I/II	National Cancer Institute (NCI), USA	MSLN-positive metastatic cancer (include lung cancer)	NCT01583686	Terminated	4-24-2012
MUC1	N/A	Anti-MUC1	20	I/II	PersonGen BioTherapeutics (Suzhou) Co., Ltd., China	MUC1 positive advanced refractory solid tumor (include NSCLC)	NCT02587689	Unknown	10-27-2015
MUC1	N/A	anti-MUC1 CAR-T cell and PD-1 knockout	60	I/II	The First Affiliated Hospital of Guangdong Pharmaceutical University, China	MUC1 positive NSCLC	NCT03525782	Recruiting	5-16-2018
TnMUC1	N/A	CAR-T-TnMUC1	112	I	Tmunity Therapeutics, USA	TnMUC1-positive relapsed/refractory multiple myeloma (include NSCLC)	NCT04025216	Recruiting	7-18-2019
CEA	N/A	Anti-CEA	75	I	Southwest Hospital of Third Millitary Medical University, China	CEA-positive cancer (include lung cancer)	NCT02349724	Unknown	1-29-2015
CEA	N/A	Anti-CEA	40	I/II	Chongqing Precision Biotech Co., Ltd, China	Relapsed and refractory CEA-positive cancer (include lung cancer)	NCT04348643	Recruiting	4-16-2020
PD-L1 and CD80/CD86	N/A	Anti-PD-L1	10	Early I	Second Xiangya Hospital of Central South University, China	Recurrent or Refractory PD-L1 positive NSCLC	NCT03060343	Unknown	2-23-2017
PD-L1	CD28/4-1BB/CD3ζ	Anti-PD-L1	22	I	Sun Yat-sen University, China	advanced PD-L1 positive NSCLC	NCT03330834	Terminated	11-6-2017
ROR1	N/A	Anti- ROR1	60	I	Fred Hutchinson Cancer Research Center, USA	Advanced ROR1-positive malignancies (include stage IV NSCLC)	NCT02706392	Recruiting	3-11-2016
HER2	CD28/4-1BB/CD3ζ	Anti-HER-2	10	I/II	Chinese PLA General Hospital, China	Chemotherapy refractory HER-2 positive advanced solid tumors (include NSCLC)	NCT01935843	Unknown	9-5-2013
HER2	N/A	Anti-HER2	0	I/II	Southwest Hospital, China	HER2-positive cancer (include lung cancer)	NCT02713984	Withdrawn	3-21-2016
HER2, MSLN, Lewis-Y, PSCA, MUC1, PD-L1	The third generation (CD28/4-1BB/OX-40/ CD3ζ)	Anti-HER2/MSLN/PSCA/MUC1/Lewis-Y/CD80/86	30	I	Second Affiliated Hospital of Guangzhou Medical University, China	Lung cancer	NCT03198052	Recruiting	6-23-2017
MAGE-A1, MAGE-A4, Muc1, GD2, MSLN	N/A	Lung cancer-specific	20	I/II	Shenzhen Geno-Immune Medical Institute, China	Lung cancer (SCLC and NSCLC)	NCT03356808	Recruiting	11-29-2017

The potential targeting antigens of NSCLC for CAR constructs in clinical trials include EGFR, MSLN, MUC1, PSCA, CEA, D-L1, CD80/CD86, ROR1, and HER2. The data of clinical trials are collected from ClinicalTrials.gov

*EGFR* epidermal growth factor receptor, *MSLN* mesothelin, *MUC1* mucin 1, *PSCA* prostate stem cell antigen, *CEA* carcinoembryonic antigen, *PD-L1* programmed death-ligand 1, *ROR1* inactive tyrosine-protein kinase transmembrane receptor, *HER2* human epidermal growth factor receptor 2, *M-D-Y* month-day-year

### EGFR

EGFR, also known as human epidermal receptor 1 (HER1), is a transmembrane glycoprotein that belongs to the ErbB receptor protein-tyrosine-kinase family. Its extracellular domain forms tumor-specific epitopes, making it an excellent target for immunotherapy. In NSCLC, over 60% of EGFR mutations are associated with tumor proliferation, neovascularization, and metastasis. Recombinant anti-EGFR CAR-T cells have specific cytolytic activity against EGFR-positive tumor cells. In one study, high levels of cytokines (IL-2, IL-4, IL-10, TNF-α, and interferon [IFN]-γ) were released 24 h after in vitro co-incubation of EGFR-positive tumor cells with anti-EGFR CAR-T cells [32]. In vivo, these CAR-T cells accounted for a high proportion of CD3^+^ CD8^+^ cytotoxic T-lymphocyte populations, lending them the ability to proliferate against NSCLC. In an ongoing phase I clinical trial at Sun Yat-sen University, C-X-C chemokine receptor (CXCR) type 5-modified anti-EGFR CAR-T cells are being assessed for efficacy and safety in treating EGFR-positive patients with advanced NSCLC (ClinicalTrials.gov identifier: NCT04153799). Of the 11 evaluated patients receiving three different doses, 2 exhibited a partial response and 5 were stable for eight months. In a phase I/II clinical study (NCT01869166) at the Chinese PLA General Hospital, advanced NSCLC patients with over 50% EGFR-positive expression on tumor cells received anti-EGFR CAR-T-cell therapy. CAR-T cells were generated from peripheral blood and stimulated in vitro for 10–13 days before treatment [33]. Patients could tolerate anti-EGFR CAR-T-cell perfusion for three to five days at a time without severe toxicity. Thus, anti-EGFR CAR-T cells may be feasible for the treatment of EGFR-positive NSCLC patients, although more clinical studies are needed to confirm these results.

### MSLN

MSLN is overexpressed in cancer cells, including in lung cancer. MSLN overexpression is strongly correlated with tumor aggressiveness, and a decreased survival rate in patients with early-stage lung adenocarcinoma [34]. In a clinical trial (NCT02414269) performed by a team from the Memorial Sloan Kettering Cancer Center, anti-MSLN inducible caspase 9-M28z (iCasp9M28z) CAR-T cells are being tested for safety and feasibility. They remarked that the amount of iCasp9M28z CAR-T cells may be over- or underestimated during its formulation. The estimated time to generate the CAR-T cells was three to six weeks. Recently, the US National Cancer Institute (NCI) terminated a phase I/II study of anti-MSLN CAR-T-cell therapy for patients with MSLN-positive metastatic lung cancer, owing to slow/insufficient accrual (NCT01583686). Intravenous management of mRNA-engineered T cells could temporarily express anti-MSLN CAR and did not disclose metastatic tumors in NSCLC. The above results demonstrate the rationale of anti-MSLN CAR-T-cell therapy for NSCLC.

### MUC1 and PSCA

MUC1 is a transmembrane glycoprotein, overexpressed in many types of cancer, including NSCLC. In an ongoing phase I/II clinical trial conducted by PersonGen BioTherapeutics (Suzhou) Co., Ltd. (NCT02587689), anti-MUC1 CAR-T cells are being used to treat advanced refractory solid tumors, including NSCLC. Another phase I/II clinical study is being conducted to assess the safety and efficacy of treatment with anti-MUC1 CAR-T cells combined with programmed cell death protein 1 (PD-1) knockout for patients with advanced NSCLC (NCT03525782). Furthermore, Tmunity Therapeutics Inc. recently registered a phase I trial (NCT04025216) to test the safety, tolerability, feasibility, and preliminary efficacy of targeting the Tn glycoform of MUC1 (TnMUC1) using CAR-T cells in TnMUC1-positive advanced cancers, including NSCLC. Clinical trials using anti-PSCA CAR-T cells for the treatment of non-lung solid tumors are ongoing (NCT02744287) or have been completed (NCT02092948). Another team constructed a variety of third-generation CAR-T cells; two of the targets are PSCA and MUC1. Currently, they are conducting a phase I study to test its safety, tolerance, and preliminary efficacy as treatment for lung cancer (NCT03198052). In a patient-derived xenograft model, anti-MUC1 CAR-T cells could not significantly suppress the growth of an NSCLC tumor mass [35]. However, a third-generation anti-PSCA CAR-T cells delayed tumor development. This indicates that PSCA-targeting CAR-T cells may comprise novel therapeutic agents for patients with NSCLC. Therefore, a combination of anti-MUC1 and anti-PSCA CAR-T cells may have a synergistic effect in combating NSCLC.

### CEA

CEA is an antigen expressed during fetal growth and development, but not in normal adult tissues and differentiated cells. CEA expression increases in certain cancers, including lung cancer, which makes it a useful tumor marker to monitor response to treatment. Preclinical evidence has established a link between serum CEA concentration and brain metastases in patients with advanced NSCLC [36]. This provided the rationale for establishing phase I clinical trials to evaluate the safety, efficacy, and maximum tolerated dose of anti-CEA CAR-T-cell therapy in CEA-positive cancers, including lung cancer (NCT02349724 and NCT04348643).

### PD-L1 and CD80/CD86

The PD-1/PD-L1 complex inhibits the cytotoxic T-cell response [37]. PD-L1 is expressed on stromal cells and tumor cells, and antibodies against PD-L1 with CAR-T cells represent a novel strategy for NSCLC [38]. The main function of the PD-1/PD-L1 pathway is to induce and maintain tolerance to self; however, tumor cells exploit this for immune escape. Recently, PD-1 antibodies have produced exciting results in an in vitro cell co-culture model, in vivo in an animal model, and in clinical trials to treat several cancers, including NSCLC [39]. Autologous CAR-T cells targeting PD-L1 and CD80/CD86 are being used for the treatment of recurrent or refractory NSCLC in an early phase I study (NCT03060343) to determine safety, tolerance, and engraftment potential. Anti-PD-L1 CAR-T-cell therapy is also being tested for safety and efficacy in advanced PD-L1-positive NSCLC patients in a phase 1 study (NCT03330834). In the latter, the T-cell activation molecules consist of CD28/4-1BB/CD3ζ and the scFv obtained from the variable regions of a PD-L1 monoclonal antibody. Undoubtedly, anti-PD-L1 CAR-T-cell therapy will be evaluated further for potential use in clinical medicine.

### ROR1

ROR1 is a tyrosine kinase-like orphan receptor, expressed in both triple-negative breast cancer (TNBC) and NSCLC [40]. ROR1-specific CAR-T-cell, engineered lentiviral vectors encoding ROR1, scFv/4-1BB/CD3ζ, and truncated EGFR molecules, allow for the elimination of ROR1 CAR-T cytotoxicity. A phase I clinical study (NCT02706392) from Fred Hutchinson Cancer Research Center was designed to assess the safety and anti-tumor effects of autologous anti-ROR1 CAR-T cells transplanted into patients with advanced, ROR1-positive, stage IV NSCLC [41]. Recently, Wallstabe et al. [40] also revealed that anti-ROR1 CAR-T cells were effective in eliminating NSCLC and TNBC cells using three-dimensional (or organoid) tumor models. Thus, anti-ROR1 CAR-T-cell therapy provides a novel strategy to treat NSCLC.

### HER2

HER2 plays an important role in the pathogenesis of various cancers [42]. This TAA is one of four members of the ErbB family of kinases [43]. It has been reported that the pan-ErbB inhibitor, afatinib, was an effective therapeutic agent in HER2-positive NSCLC, suggesting that HER2 can be considered as a therapeutic target for NSCLC [44]. Researchers have developed anti-HER2 CAR-T cells with 4-1BB and CD3ζ signaling domains, and initiated a phase I/II study to demonstrate its safety and feasibility in treating HER2-positive solid tumors, including NSCLC (NCT01935843). In this trial, anti-HER2 CAR-T cells would be infused for three days in patients showing no unacceptable toxicity. Another phase I/II clinical research study of CAR-T cells targeting HER2-positive cancers (including lung cancer) has been withdrawn to change the CAR structure due to the safety considerations (NCT02713984). Thus, to date, no clinical results have been reported for anti-HER2 CAR-T-cell therapy for NSCLC.

### Other targeted antigens

Other antigens (single or combined) that are being targeted using CAR-T cells in clinical trials on lung cancer patients include ganglioside GD2, melanoma-associated antigen (MAGE)-A1, MAGE-A4, and Lewis-Y antigen (NCT03198052 and NCT03356808). Finally, several co-identified TAAs (such as HER2 and IL-13Rα2) are yet to be tested as targets for CAR-T-cell treatment of NSCLC.

## Current challenges and strategies of CAR-T-cell therapy in NSCLC

The general strategy in CAR-T-cell therapy is the utilization of gene transfer technology to reprogram patients’ T cells to express a CAR that can bind to common antigens [45]. After transfection of autologous or allogeneic peripheral blood T cells with the CAR complex, transfected cells are injected into a patient as cytotoxic agents, attacking cancer cells [46]. The clinical infusion process is illustrated in Supplementary Fig. 1. CAR-T cells recognize TAAs through the scFv. Beneficial outcomes have been reported in a clinical study of CAR-T-cell treatment in B-cell malignant hematological disease [16, 17]. Unlike in the treatment of hematologic malignancies, CAR-T-cell treatment of solid tumors (such as NSCLC) has had limited success thus far [47]. Barriers to success include: (1) on-target/off-tumor toxicity; (2) neurological toxicity; (3) cytokine release syndrome (CRS); (4) a paucity of tumor-specific antigens; (5) an immunosuppressive tumor microenvironment (TME); (6) low levels of infiltration into tumor tissue; and (7) tumor antigen escape. See Supplementary Fig. 2 for a diagram of current challenges for CAR-T-cell therapy in NSCLC and strategies to overcome these.

### On-target/off-tumor toxicity

Although CAR-T cells provide a promising approach for treating NSCLC, their toxicity in clinical practice remains a concern. On-target/off-tumor toxicity is caused when CAR-T cells bind to a targeted antigen on normal (off-tumor) cells; this happens because many TAAs are not tumor-specific. The extent of the on-target/off-tumor effect differs between patients and antigens; it may result in disorders affecting various organ systems (including the pulmonary, hematologic, and gastrointestinal systems) and even be life-threatening [48]. In order to minimize these risks, CARs should be as selective as possible. This can be achieved by selecting safer antigens (such as EGFR variant III and aberrantly glycosylated antigens) [49, 50]. Such antigens still need to be explored for use in NSCLC in upcoming clinical trials.

### Neurological toxicity

Nervous system toxicity is a common adverse reaction to CAR-T-cell treatment, and includes confusion and seizures [51]. The NCI recommends high-dose corticosteroids for the treatment of such grade 3 neurotoxicities persisting for ≥ 24 h, and for all grade 4 neurotoxicities [52]. An immunoglobulin E-mediated clinical allergic reaction following CAR-T-cell infusion has been reported [53]. Tumor lysis syndrome has also been described in certain patients undergoing CAR-T-cell therapy [52]. Further understanding and in-depth analysis of the pathophysiology of these toxicities is needed to better define the choice of preventive measures and to calculate better expanded treatment with regard to neurological toxicities [54]. Thus, insight into the pathophysiology will be a vital factor to inhibit systemic neurotoxicity in future developments of CAR-T-cell treatment.

### CRS

CRS is a major adverse effect of CAR-T-cell therapy, causing dangerous and even life-threatening toxicity [14, 52]. It is characterized by the release of a variety of inflammatory cytokines by the infused T cells upon antigen recognition, resulting in a significant increase in the expression levels of TNF-α, C-reactive protein, IL-2, IL-6, IL-8, and IFN-γ [55]. Downstream complications include fever, fatigue, anorexia, hypotension, multi-organ dysfunction, and even sudden death due to the cytokine storm [46, 52]. The diagnosis and management of CRS is crucial to patient health, and there is a wealth of evidence supporting the use of inhibitors of the IL-6 pathway, such as tocilizumab or siltuximab, to treat CRS [56]. Infliximab is another cytokine inhibitor that should be considered as the TNF-α inhibitor, [57]. It is important for clinical researchers to fully understand the biology of the syndrome, to propose appropriate solutions for its prevention by supplying cytokine inhibitors to reduce inflammation. Another important issue is the identification of CRS biomarkers. Teachey et al. [58] discovered that a combination of three cytokines (IFN-γ, soluble IL-1 receptor agonist, and glycoprotein subunit 130) were accurate predictors of CRS within one month after anti-CD19 CAR-T-cell infusion for lymphoblastic leukemia. These results should be used to guide prediction, diagnosis, and management of CRS in future research of CAR-T-cell therapy for NSCLC.

### Paucity of tumor-specific antigens

Currently, the effective way to treat solid tumors with CAR-T cells is to identify tumor-specific cell-surface antigens [59]. However, the heterogeneity in biological structure of solid tumors is an important limiting factor [60]. Therefore, there is an urgent need to discover new TAAs to improve clinical utility and safety, as well as to maintain the anti-tumor activity of CAR-T cells. One approach would be to treat patients with agents that increase expression of the target antigen. In one pre-clinical study, this approach was followed, using all-*trans*-retinoic acid to increase the expression of folate receptor beta in acute myeloid leukemia [61]. Alternatively, engineered CAR can be used to enhance T-cell activity against antigens present at lower densities. The commonly attempted modification to date has been to decrease the affinity of the scFv for preferential identification of its target. One group have demonstrated that reducing the affinity of anti-EGFR CAR-T cells for their antigens causes T cells to recognize malignant cells preferentially, as they contain higher levels of the antigen than normal cells do [62]. However, it remains unclear at what level this enhancement will reach a plateau. In addition, as CAR-T-cell technology is developed to target different TAAs in hematological and solid tumors, research is required to uncover the mechanisms by which tumor cells express specific antigens, which will assist in the optimization of adoptive immunotherapy for NSCLC.

### Immunosuppressive TME

The clinical efficacy of CAR-T cells in the treatment of NSCLC is impeded by TME-related factors such as hypoxia, lack of arginine or tryptophan, suppression of tumor-derived cytokines, and inhibition of T cells [31]. Studies have demonstrated that the rapid loss of CAR-T-cell function limits its therapeutic role in the immunosuppressive TME [46]. Therefore, a useful avenue to explore is the optimization of CAR-T cell therapy in combination with other NSCLC treatments. The effect of CAR-T-cell therapy can be enhanced by combining it with immune checkpoint inhibitors; the latter reduces immunosuppression of CAR-T cells by creating a more favorable TME. Many ongoing clinical trials are investigating the binding of CAR-T cells to checkpoint inhibitors to reduce immunosuppression [29, 63]. CAR-T-cell therapy can also be enhanced by expressing immune-related factors. IL-12 is an immunomodulatory cytokine that stimulates the immune response via natural killer cells and T cells. Yeku et al. [64] demonstrated that IL-12-armored CAR-T cells can exert their anti-tumor function through overcoming the immunosuppressive TME. Zhou et al. [65] reported that IL-7/IL-5 yielded a superior anti-tumor function through improving CAR-T-cell proliferation, reducing CAR-T-cell apoptosis, and resisting the immunosuppressive TME. Therefore, CAR-T-cells engineered to co-express such immune-related factors should also be investigated for their potential to treat NSCLC.

### Low levels of infiltration into tumor tissue

NSCLC has unique histopathological features such as a high blood vessel concentration, poor structural integrity, and extensive leakage of blood vessels [9]. These characteristics lead to enhanced permeability and retention of lipid particles and macromolecular substances in NSCLC. A large number of tumor infiltrating lymphocytes and extensive infiltration have been considered as one of the main indicators for patient diagnosis with NSCLC [66–68]. Some studies have revealed that infiltration of CD3^+^ T cells, CD8^+^ T cells, and B lymphocytes were closely related to the presence of high endothelial venules. Overexpression of endothelin B receptor and down-regulation of intercellular adhesion molecule 1 can also block the homing of T cells, which directly reduces the efficiency of tumor immunotherapy [69]. Therefore, attracting more CAR-T cells into tumor tissues will increase efficiency.

Chemokines belong to a large class of cytokine subfamily. Most of the chemokines have chemotactic functions and guide cell migration by binding to their homologous receptors [70]. Tumor, stromal, and immune cells express chemokines [71]. At present, the chemokine system (including chemokines and their corresponding receptors) has become a new potential target for cancer immunotherapy [71, 72]. A variety of methods have been developed to target chemokine ligands for neutralization, or to modify T cells to overexpress chemokine receptors that express homologous ligands in the TME [73]. Some studies have confirmed this principle of bypassing the systemic circulation, allowing CAR-T cells to express chemokine receptors such as CXCR 2 (the receptor to CXC ligand 1) and C–C chemokine receptor 4 (the receptor to CC ligand 17) [59, 74]. Therefore, blocking tumor-derived chemokines and overexpressing chemokine receptors is an ideal strategy for CAR-T-cell immunotherapy in mediating cell migration to improve CAR-T cell infiltration.

### Tumor antigen escape

Normally, T cells expressing two or more independent CAR molecules have more effective anti-tumor functions than those of T cells expressing a single CAR molecule [75]. However, the difference is that T cells can be equipped with two or more CARs that recognize different TAAs. Antigen escape remains a major mechanism of relapse and is a key barrier for expanding the use of CAR-T cells towards solid cancers with their more diverse surface antigen repertoires [60]. For some malignancies, it can be difficult to determine whether CAR-T-cell therapy against a specific combination of TAAs is safe and effective. Recruiting or enhancing the endogenous anti-tumor immune response may enable targeting of a broader range of tumors by involving many different effector cell types. Potential methods for overcoming this challenge include enabling CAR-T cells to specifically recognize multiple antigens and respond to lower levels of target cell antigens [75]. It is speculated that mutations in tumor cells may produce new human leukocyte antigens (HLAs), which may induce specific endogenous T-cell responses that enhance the overall anti-tumor effect gained by immune checkpoint inhibitors [76]. Nevertheless, this may not be sufficient to prevent tumor growth, and these tumors cannot be recognized by CARs or endogenous T cells. Sotillo and colleagues [77] demonstrated a unique mechanism by which tumors can mediate immune escape. Anti-CD19 CAR-T-cell therapy caused selection of pre-existing variants of CD19 in certain patients, thereby altering the target epitope beyond recognition by the CAR. These findings are important for developing strategies to overcome the risk of tumor antigen escape in immune-based anti-tumor therapies, including for NSCLC.

## Future perspectives for CAR-T-cell therapy in NSCLC

As a novel strategy for the treatment of NSCLC, CAR-T cell therapy has undergone great progress and has entered a stage of rapid development [18]. Although it has certain side effects and potential risks [52], it exhibits a more potent target-binding ability, longer duration of in vivo efficacy, and exerts a more rapid therapeutic effect on NSCLC than conventional treatment does. Indeed, CARs have overcome the initial limitations of cancer therapy, such as HLA restriction and major histocompatibility complex class I down-regulation; however, they are currently limited by other tumor escape methods [75]. Conventional CARs have also been modified to improve their safety and efficacy in the form of dual-target, multi-target, drug-inducible, switchable, inhibitory, and universal CARs [21, 46, 78, 79]. These CARs are being studied in both animal models and clinical trials in the attempt to mitigate tumor antigen heterogeneity, and may eventually constitute the next generation of CAR-T cells [22, 74]. The ultimate goal of the development of CAR-T cells is to cure cancers such as NSCLC.

## Conclusion

Many obstacles remain in the application of CAR-T-cell therapy in NSCLC, especially the heterogeneity of antigens expressed by tumor cells. The mechanisms of tumor resistance to such therapy need to be better understood and accurate predictors of the response to immunotherapy need to be discovered. The next steps will be to better identify patients with a primary or acquired risk of resistance to CAR-T-cell therapy, as well as to develop suitable combined therapies, utilizing an increasingly evaluative and systematic bioinformatics approach. This will allow for individualized patient care based on precision medicine. Taken together, research on CAR-T cells for the treatment of NSCLC is underway and has yielded promising preliminary results, both in basic and pre-clinical medicine. Therefore, more basic research and clinical trials are warranted.

### Electronic supplementary material

Below is the link to the electronic supplementary material.

Supplementary file1 (PDF 2252 kb)

## Data Availability

Please contact corresponding author for data requests.

## Abbreviations

CAR: Chimeric antigen receptor
CEA: Carcinoembryonic antigen
CRS: Cytokine release syndrome
CXCR: C-X-C chemokine receptor
EGFR: Epidermal growth factor receptor
GITR: Glucocorticoid-induced TNF receptor-related protein
HER1: Human epidermal receptor 1
HER2: Human epidermal growth factor receptor 2
HLA: Human leukocyte antigen
iCasp9M28z: Inducible caspase 9-M28z
ICOS: Inducible costimulator
IFN: Interferon
IL: Interleukin
IL-2Rβ: IL-2 receptor β
JAK: Janus kinase
MAGE: Melanoma-associated antigen
MSLN: Mesothelin
MUC1: Mucin 1
NCI: National Cancer Institute
NFAT: Nuclear factor of activated T cell
NSCLC: Non-small-cell lung cancer
PD-1: Programmed cell death protein 1
PD-L1: Programmed death ligand 1
PSCA: Prostate stem cell antigen
ROR1: Inactive tyrosine-protein kinase transmembrane receptor
scFv: Single chain variable fragment
STAT: Signal transducer and activator of transcription
TAA: Tumor-associated antigen
TM: Transmembrane
TME: Tumor microenvironment
TNBC: Triple-negative breast cancer
TNF: Tumor necrosis factor
TnMUC1: Tn glycoform of MUC1
TRUCK: T cells redirected for universal cytokine-mediated killing

## References

[1] Hirsch FR, Scagliotti GV, Mulshine JL, Kwon R, Curran WJ, Wu Y-L, Paz-Ares L (2017). Lung cancer: current therapies and new targeted treatments. Lancet.

[2] Siegel RL, Miller KD, Jemal A (2020). Cancer statistics. CA Cancer J Clin.

[3] Allemani C, Matsuda T, Di Carlo V, Harewood R, Matz M, Niksic M, Bonaventure A, Valkov M, Johnson CJ, Esteve J, Ogunbiyi OJ, Azevedo ESG, Chen WQ, Eser S, Engholm G, Stiller CA, Monnereau A, Woods RR, Visser O, Lim GH, Aitken J, Weir HK, Coleman MP, Group CW (2018). Global surveillance of trends in cancer survival 2000–14 (CONCORD-3): analysis of individual records for 37 513 025 patients diagnosed with one of 18 cancers from 322 population-based registries in 71 countries. Lancet.

[4] Chen Z, Fillmore CM, Hammerman PS, Kim CF, Wong K-K (2014). Non-small-cell lung cancers: a heterogeneous set of diseases. Nat Rev Cancer.

[5] Gandhi L, Rodríguez-Abreu D, Gadgeel S, Esteban E, Felip E, De Angelis F, Domine M, Clingan P, Hochmair MJ, Powell SF, Cheng SYS, Bischoff HG, Peled N, Grossi F, Jennens RR, Reck M, Hui R, Garon EB, Boyer M, Rubio-Viqueira B, Novello S, Kurata T, Gray JE, Vida J, Wei Z, Yang J, Raftopoulos H, Pietanza MC, Garassino MC (2018). Pembrolizumab plus chemotherapy in metastatic non–small-cell lung cancer. N Engl J Med.

[6] Leventakos K, Mansfield AS (2016). Advances in the treatment of non-small cell lung cancer: focus on nivolumab, pembrolizumab, and atezolizumab. BioDrugs.

[7] Hellmann MD, Paz-Ares L, Bernabe Caro R, Zurawski B, Kim SW, Carcereny Costa E, Park K, Alexandru A, Lupinacci L, de la Mora JE, Sakai H, Albert I, Vergnenegre A, Peters S, Syrigos K, Barlesi F, Reck M, Borghaei H, Brahmer JR, O'Byrne KJ, Geese WJ, Bhagavatheeswaran P, Rabindran SK, Kasinathan RS, Nathan FE, Ramalingam SS (2019). Nivolumab plus ipilimumab in advanced non-small-cell lung cancer. N Engl J Med.

[8] Zhang C, Leighl NB, Wu YL, Zhong WZ (2019). Emerging therapies for non-small cell lung cancer. J Hematol Oncol.

[9] Herbst RS, Morgensztern D, Boshoff C (2018). The biology and management of non-small cell lung cancer. Nature.

[10] Parikh K, Huether R, White K, Hoskinson D, Beaubier N, Dong H, Adjei AA, Mansfield AS (2020). Tumor mutational burden from tumor-only sequencing compared with germline subtraction from paired tumor and normal specimens. JAMA Netw Open.

[11] Ozaki Y, Muto S, Takagi H, Watanabe M, Inoue T, Fukuhara M, Yamaura T, Okabe N, Matsumura Y, Hasegawa T, Ohsugi J, Hoshino M, Shio Y, Tanaka D, Nanamiya H, Imai JI, Isogai T, Watanabe S, Suzuki H (2020). Tumor mutation burden and immunological, genomic, and clinicopathological factors as biomarkers for checkpoint inhibitor treatment of patients with non-small-cell lung cancer. Cancer Immunol Immunother CII.

[12] Nadal E, Massuti B, Domine M, Garcia-Campelo R, Cobo M, Felip E (2019). Immunotherapy with checkpoint inhibitors in non-small cell lung cancer: insights from long-term survivors. Cancer Immunol Immunother CII.

[13] Barham W, Gicobi JK, Yan Y, Dronca RS, Dong H (2019). Paradox-driven adventures in the development of cancer immunology and immunotherapy. Genes Dis.

[14] D'Aloia MM, Zizzari IG, Sacchetti B, Pierelli L, Alimandi M (2018). CAR-T cells: the long and winding road to solid tumors. Cell Death Dis.

[15] Kim DW, Cho JY (2020). Recent advances in allogeneic CAR-T cells. Biomolecules.

[16] Ying Z, Huang XF, Xiang X, Liu Y, Kang X, Song Y, Guo X, Liu H, Ding N, Zhang T, Duan P, Lin Y, Zheng W, Wang X, Lin N, Tu M, Xie Y, Zhang C, Liu W, Deng L, Gao S, Ping L, Wang X, Zhou N, Zhang J, Wang Y, Lin S, Mamuti M, Yu X, Fang L, Wang S, Song H, Wang G, Jones L, Zhu J, Chen SY (2019). A safe and potent anti-CD19 CAR T cell therapy. Nat Med.

[17] Patel AJ, Richter A, Drayson MT, Middleton GW (2020). The role of B lymphocytes in the immuno-biology of non-small-cell lung cancer. Cancer Immunol Immunother CII.

[18] Doroshow DB, Sanmamed MF, Hastings K, Politi K, Rimm DL, Chen L, Melero I, Schalper KA, Herbst RS (2019). Immunotherapy in non-small cell lung cancer: facts and hopes. Clin Cancer Res.

[19] Hu Z, Zheng X, Jiao D, Zhou Y, Sun R, Wang B, Tian Z, Wei H (2020). LunX-CAR T cells as a targeted therapy for non-small cell lung cancer. Mol Ther Oncol.

[20] Srivastava S, Riddell SR (2015). Engineering CAR-T cells: design concepts. Trends Immunol.

[21] MacKay M, Afshinnekoo E, Rub J, Hassan C, Khunte M, Baskaran N, Owens B, Liu L, Roboz GJ, Guzman ML, Melnick AM, Wu S, Mason CE (2020). The therapeutic landscape for cells engineered with chimeric antigen receptors. Nat Biotechnol.

[22] Tokarew N, Ogonek J, Endres S, von Bergwelt-Baildon M, Kobold S (2019). Teaching an old dog new tricks: next-generation CAR T cells. Br J Cancer.

[23] Firor AE, Jares A, Ma Y (2015). From humble beginnings to success in the clinic: chimeric antigen receptor-modified T-cells and implications for immunotherapy. Exp Biol Med (Maywood).

[24] Sadelain M, Brentjens R, Riviere I (2013). The basic principles of chimeric antigen receptor design. Cancer Discov.

[25] Guedan S, Ruella M, June CH (2019). Emerging cellular therapies for cancer. Annu Rev Immunol.

[26] Chmielewski M, Abken H (2015). TRUCKs: the fourth generation of CARs. Exp Opin Biol Ther.

[27] Zhao L, Cao YJ (2019). Engineered T cell therapy for cancer in the clinic. Front Immunol.

[28] Yong CSM, Dardalhon V, Devaud C, Taylor N, Darcy PK, Kershaw MH (2017). CAR T-cell therapy of solid tumors. Immunol Cell Biol.

[29] Li J, Li W, Huang K, Zhang Y, Kupfer G, Zhao Q (2018). Chimeric antigen receptor T cell (CAR-T) immunotherapy for solid tumors: lessons learned and strategies for moving forward. J Hematol Oncol.

[30] Titov A, Valiullina A, Zmievskaya E, Zaikova E, Petukhov A, Miftakhova R, Bulatov E, Rizvanov A (2020). Advancing CAR T-cell therapy for solid tumors: lessons learned from lymphoma treatment. Cancers.

[31] Kiesgen S, Chicaybam L, Chintala NK, Adusumilli PS (2018). Chimeric antigen receptor (CAR) T-cell therapy for thoracic malignancies. J Thorac Oncol.

[32] Ke EE, Wu YL (2016). EGFR as a pharmacological target in EGFR-mutant non-small-cell lung cancer: where do we stand now?. Trends Pharmacol Sci.

[33] Liu Y, Guo Y, Wu Z, Feng K, Tong C, Wang Y, Dai H, Shi F, Yang Q, Han W (2020). Anti-EGFR chimeric antigen receptor-modified T cells in metastatic pancreatic carcinoma: a phase I clinical trial. Cytotherapy.

[34] Kachala SS, Bograd AJ, Villena-Vargas J, Suzuki K, Servais EL, Kadota K, Chou J, Sima CS, Vertes E, Rusch VW, Travis WD, Sadelain M, Adusumilli PS (2014). Mesothelin overexpression is a marker of tumor aggressiveness and is associated with reduced recurrence-free and overall survival in early-stage lung adenocarcinoma. Clin Cancer Res.

[35] Wei X, Lai Y, Li J, Qin L, Xu Y, Zhao R, Li B, Lin S, Wang S, Wu Q, Liang Q, Peng M, Yu F, Li Y, Zhang X, Wu Y, Liu P, Pei D, Yao Y, Li P (2017). PSCA and MUC1 in non-small-cell lung cancer as targets of chimeric antigen receptor T cells. Oncoimmunology.

[36] Grunnet M, Sorensen JB (2012). Carcinoembryonic antigen (CEA) as tumor marker in lung cancer. Lung Cancer (Amsterdam, Netherlands).

[37] Johnson RMG, Wen T, Dong H (2020). Bidirectional signals of PD-L1 in T cells that fraternize with cancer cells. Nat Immunol.

[38] Liu M, Wang X, Li W, Yu X, Flores-Villanueva P, Xu-Monette ZY, Li L, Zhang M, Young KH, Ma X, Li Y (2020). Targeting PD-L1 in non-small cell lung cancer using CAR T cells. Oncogenesis.

[39] Takamori S, Toyokawa G, Takada K, Shoji F, Okamoto T, Maehara Y (2018). Combination therapy of radiotherapy and anti-PD-1/PD-L1 treatment in non-small-cell lung cancer: a mini-review. Clin Lung Cancer.

[40] Wallstabe L, Gottlich C, Nelke LC, Kuhnemundt J, Schwarz T, Nerreter T, Einsele H, Walles H, Dandekar G, Nietzer SL, Hudecek M (2019). ROR1-CAR T cells are effective against lung and breast cancer in advanced microphysiologic 3D tumor models. JCI Insight.

[41] Specht JM, Lee S, Turtle CJ, Berger C, Baladrishnan A, Srivastava S, Voillet V, Veatch J, Gooley T, Mullane E, Chaney C, Rader C, Pierce RH, Gottardo R, Maloney DG, Riddell SR (2018). A phase I study of adoptive immunotherapy for advanced ROR1+malignancies with defined subsets of autologous T cells expressing a ROR1-specific chimeric antigen receptor (ROR1-CAR). Cancer Res.

[42] Yan M, Schwaederle M, Arguello D, Millis SZ, Gatalica Z, Kurzrock R (2015). HER2 expression status in diverse cancers: review of results from 37,992 patients. Cancer Metastasis Rev.

[43] Roskoski R (2014). The ErbB/HER family of protein-tyrosine kinases and cancer. Pharmacol Res.

[44] Hirsh V (2015). Next-generation covalent irreversible kinase inhibitors in NSCLC: focus on afatinib. BioDrugs.

[45] June CH, O'Connor RS, Kawalekar OU, Ghassemi S, Milone MC (2018). CAR T cell immunotherapy for human cancer. Science.

[46] Rafiq S, Hackett CS, Brentjens RJ (2020). Engineering strategies to overcome the current roadblocks in CAR T cell therapy. Nat Rev Clin Oncol.

[47] Fuca G, Reppel L, Landoni E, Savoldo B, Dotti G (2020). Enhancing chimeric antigen receptor T-cell efficacy in solid tumors. Clin Cancer Res.

[48] Bonifant CL, Jackson HJ, Brentjens RJ, Curran KJ (2016). Toxicity and management in CAR T-cell therapy. Mol Ther Oncolytics.

[49] Morgan RA, Johnson LA, Davis JL, Zheng Z, Woolard KD, Reap EA, Feldman SA, Chinnasamy N, Kuan CT, Song H, Zhang W, Fine HA, Rosenberg SA (2012). Recognition of glioma stem cells by genetically modified T cells targeting EGFRvIII and development of adoptive cell therapy for glioma. Hum Gene Ther.

[50] Posey AD, Schwab RD, Boesteanu AC, Steentoft C, Mandel U, Engels B, Stone JD, Madsen TD, Schreiber K, Haines KM, Cogdill AP, Chen TJ, Song D, Scholler J, Kranz DM, Feldman MD, Young R, Keith B, Schreiber H, Clausen H, Johnson LA, June CH (2016). Engineered CAR T Cells targeting the cancer-associated tn-glycoform of the membrane mucin MUC1 control adenocarcinoma. Immunity.

[51] Wang Z, Guo Y, Han W (2017). Current status and perspectives of chimeric antigen receptor modified T cells for cancer treatment. Protein Cell.

[52] Brudno JN, Kochenderfer JN (2016). Toxicities of chimeric antigen receptor T cells: recognition and management. Blood.

[53] Maus MV, Haas AR, Beatty GL, Albelda SM, Levine BL, Liu X, Zhao Y, Kalos M, June CH (2013). T cells expressing chimeric antigen receptors can cause anaphylaxis in humans. Cancer Immunol Res.

[54] Gauthier J, Yakoub-Agha I (2017). Chimeric antigen-receptor T-cell therapy for hematological malignancies and solid tumors: clinical data to date, current limitations and perspectives. Curr Res Transl Med.

[55] Abken H (2017). Driving CARs on the highway to solid cancer: some considerations on the adoptive therapy with CAR T cells. Hum Gene Ther.

[56] Chen F, Teachey DT, Pequignot E, Frey N, Porter D, Maude SL, Grupp SA, June CH, Melenhorst JJ, Lacey SF (2016). Measuring IL-6 and sIL-6R in serum from patients treated with tocilizumab and/or siltuximab following CAR T cell therapy. J Immunol Methods.

[57] Lee DW, Gardner R, Porter DL, Louis CU, Ahmed N, Jensen M, Grupp SA, Mackall CL (2014). Current concepts in the diagnosis and management of cytokine release syndrome. Blood.

[58] Teachey DT, Lacey SF, Shaw PA, Melenhorst JJ, Maude SL, Frey N, Pequignot E, Gonzalez VE, Chen F, Finklestein J, Barrett DM, Weiss SL, Fitzgerald JC, Berg RA, Aplenc R, Callahan C, Rheingold SR, Zheng Z, Rose-John S, White JC, Nazimuddin F, Wertheim G, Levine BL, June CH, Porter DL, Grupp SA (2016). Identification of predictive biomarkers for cytokine release syndrome after chimeric antigen receptor T-cell therapy for acute lymphoblastic leukemia. Cancer Discov.

[59] Martinez M, Moon EK (2019). CAR T cells for solid tumors: new strategies for finding, infiltrating, and surviving in the tumor microenvironment. Front Immunol.

[60] Kailayangiri S, Altvater B, Wiebel M, Jamitzky S, Rossig C (2020). Overcoming heterogeneity of antigen expression for effective CAR T cell targeting of cancers. Cancers.

[61] Lynn RC, Poussin M, Kalota A, Feng Y, Low PS, Dimitrov DS, Powell DJ (2015). Targeting of folate receptor beta on acute myeloid leukemia blasts with chimeric antigen receptor-expressing T cells. Blood.

[62] Caruso HG, Hurton LV, Najjar A, Rushworth D, Ang S, Olivares S, Mi T, Switzer K, Singh H, Huls H, Lee DA, Heimberger AB, Champlin RE, Cooper LJ (2015). Tuning sensitivity of CAR to EGFR density limits recognition of normal tissue while maintaining potent antitumor activity. Cancer Res.

[63] Prelaj A, Tay R, Ferrara R, Chaput N, Besse B, Califano R (2019). Predictive biomarkers of response for immune checkpoint inhibitors in non–small-cell lung cancer. Eur J Cancer (Oxford England: 1990).

[64] Yeku OO, Purdon TJ, Koneru M, Spriggs D, Brentjens RJ (2017). Armored CAR T cells enhance antitumor efficacy and overcome the tumor microenvironment. Sci Rep.

[65] Zhou J, Jin L, Wang F, Zhang Y, Liu B, Zhao T (2019). Chimeric antigen receptor T (CAR-T) cells expanded with IL-7/IL-15 mediate superior antitumor effects. Protein Cell.

[66] He Y, Yu H, Rozeboom L, Rivard CJ, Ellison K, Dziadziuszko R, Suda K, Ren S, Wu C, Hou L, Zhou C, Hirsch FR (2017). LAG-3 protein expression in non-small cell lung cancer and its relationship with PD-1/PD-L1 and tumor-infiltrating lymphocytes. J Thorac Oncol.

[67] O'Brien SM, Klampatsa A, Thompson JC, Martinez MC, Hwang WT, Rao AS, Standalick JE, Kim S, Cantu E, Litzky LA, Singhal S, Eruslanov EB, Moon EK, Albelda SM (2019). Function of human tumor-infiltrating lymphocytes in early-stage non-small cell lung cancer. Cancer Immunol Res.

[68] Zhang J, Endres S, Kobold S (2019). Enhancing tumor T cell infiltration to enable cancer immunotherapy. Immunotherapy.

[69] Vedvyas Y, McCloskey JE, Yang Y, Min IM, Fahey TJ, Zarnegar R, Hsu YS, Hsu JM, Besien KV, Gaudet I, Law P, Kim NJ, Hofe EV, Jin MM (2019). Manufacturing and preclinical validation of CAR T cells targeting ICAM-1 for advanced thyroid cancer therapy. Sci Rep.

[70] Mollica Poeta V, Massara M, Capucetti A, Bonecchi R (2019). Chemokines and chemokine receptors: new targets for cancer immunotherapy. Front Immunol.

[71] Nagarsheth N, Wicha MS, Zou W (2017). Chemokines in the cancer microenvironment and their relevance in cancer immunotherapy. Nat Rev Immunol.

[72] Do HTT, Lee CH, Cho J (2020). Chemokines and their receptors: multifaceted roles in cancer progression and potential value as cancer prognostic markers. Cancers.

[73] Benmebarek MR, Karches CH, Cadilha BL, Lesch S, Endres S, Kobold S (2019). Killing mechanisms of chimeric antigen receptor (CAR) T cells. Int J Mol Sci.

[74] Tian Y, Li Y, Shao Y, Zhang Y (2020). Gene modification strategies for next-generation CAR T cells against solid cancers. J Hematol Oncol.

[75] Majzner RG, Mackall CL (2018). Tumor antigen escape from CAR T-cell therapy. Cancer Discov.

[76] Failing JJ, Aubry MC, Mansfield AS (2020). Human leukocyte antigen expression in paired primary lung tumors and brain metastases in non-small cell lung cancer. Cancer Immunol Immunother CII.

[77] Sotillo E, Barrett DM, Black KL, Bagashev A, Oldridge D, Wu G, Sussman R, Lanauze C, Ruella M, Gazzara MR, Martinez NM, Harrington CT, Chung EY, Perazzelli J, Hofmann TJ, Maude SL, Raman P, Barrera A, Gill S, Lacey SF, Melenhorst JJ, Allman D, Jacoby E, Fry T, Mackall C, Barash Y, Lynch KW, Maris JM, Grupp SA, Thomas-Tikhonenko A (2015). Convergence of acquired mutations and alternative splicing of CD19 enables resistance to CART-19 immunotherapy. Cancer Discov.

[78] Lindner SE, Johnson SM, Brown CE, Wang LD (2020). Chimeric antigen receptor signaling: functional consequences and design implications. Sci Adv.

[79] Feldmann A, Arndt C, Koristka S, Berndt N, Bergmann R, Bachmann MP (2019). Conventional CARs versus modular CARs. Cancer Immunol Immunother CII.

