# Supplementary material for: Chimeric antigen receptor (CAR)-T-cell therapy in non-small-cell lung cancer (NSCLC): current status and future perspectives
Source: Cancer Immunol Immunother. 2020 Oct 6;70(3):619–31. doi: 10.1007/s00262-020-02735-0 (PMC7907037; doi:10.1007/s00262-020-02735-0)
Supplement: Supplementary file 1 — Supplementary file1 (PDF 2252 kb) [file 262_2020_2735_MOESM1_ESM.pdf]

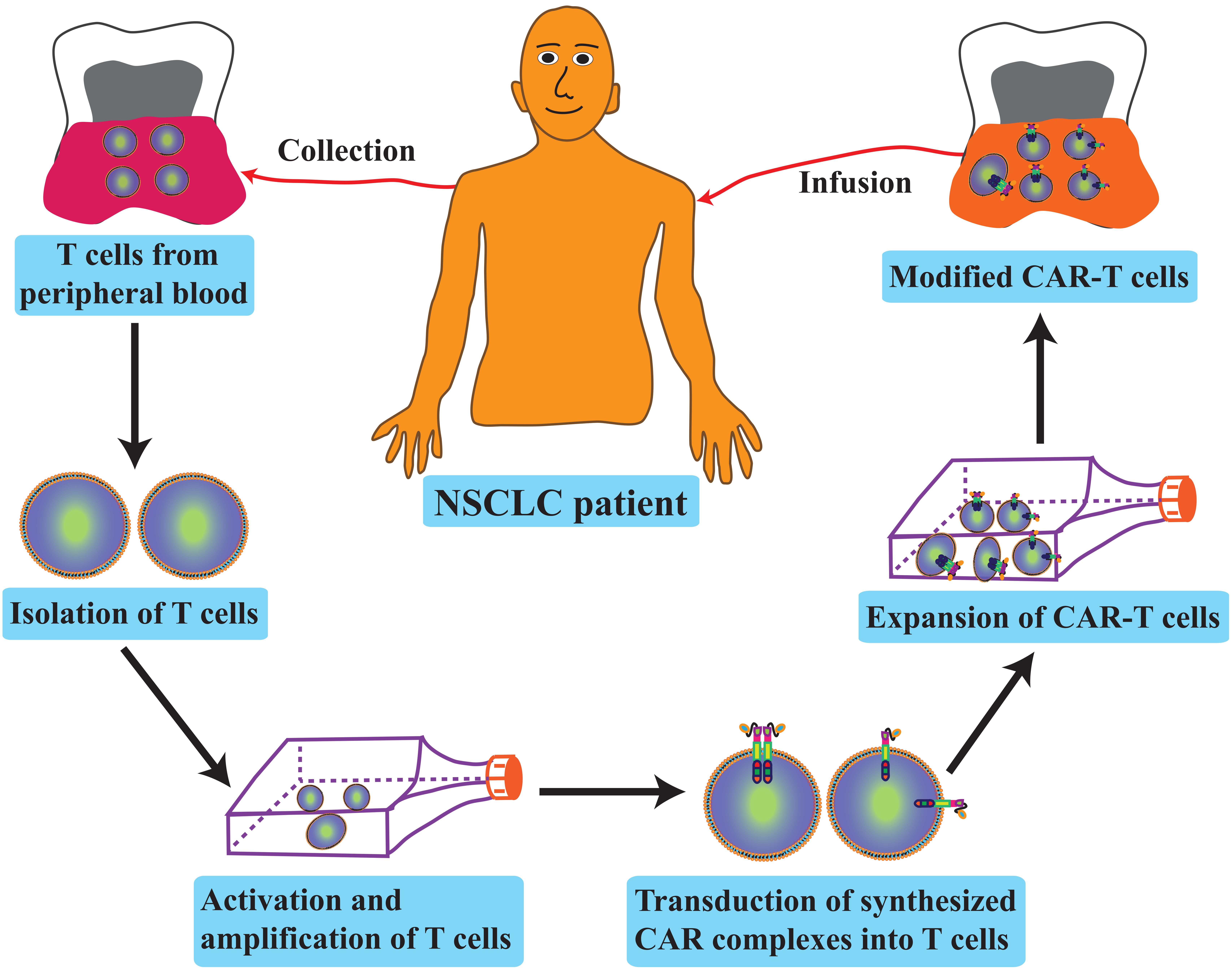

Supplementary Fig. 1. The clinical infusion process of CAR-T cell in NSCLC patient. Firstly, peripheral blood is collected from NSCLC patient. Then, T lymphocytes are isolated, and T cells are activated and amplified. The synthesized CAR complexes are transduced into T cells using lentivirus carrier. Subsequently, genetically engineered CAR-T cells are expanded. Eventually, these CAR-T cells are injected into a patient as cytotoxic agents attacking cancer cells.

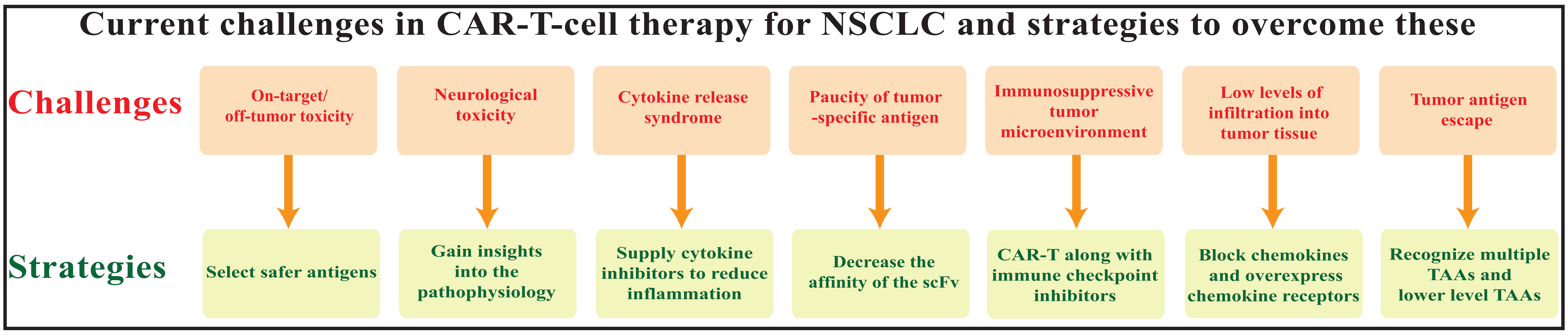

Supplementary Fig. 2. The diagram of current challenges in CAR-T-cell therapy for NSCLC and strategies to overcome these. The strategies for overcoming the current barriers include: (1) select safer antigens to overcome on-target/off-tumor toxicity; (2) gain insights into the pathophysiology of neurological toxicity to reduce adverse events; (3) supply cytokine inhibitors to reduce inflammation to solve the issue of cytokine release syndrome; (4) decrease the affinity of the scFv to address the paucity of tumor-specific antigens; (5) use CAR-T along with immune checkpoint inhibitors to reduce an immunosuppressive tumor microenvironment; (6) block chemokines and overexpress chemokine receptors to solve the issue of low levels of infiltration into tumor tissue; and (7) recognize multiple TAAs and lower level TAAs to decrease tumor antigen escape.
